# Genomic evolution of *Salmonella* Dublin in cattle and humans in the United States

**DOI:** 10.1128/aem.00689-25

**Published:** 2025-08-19

**Authors:** Sophia M. Kenney, Nkuchia M. M'ikanatha, Erika Ganda

**Affiliations:** 1 Department of Animal Science, The Pennsylvania State University 227772, University Park, Pennsylvania, USA; 2 Molecular, Cellular, and Integrative Biosciences Graduate Program, Huck Institutes of the Life Sciences, The Pennsylvania State University 124474, University Park, Pennsylvania, USA; 3 One Health Microbiome Center, Huck Institutes of the Life Sciences, The Pennsylvania State University 124474, University Park, Pennsylvania, USA; 4 Department of Food Science, The Pennsylvania State University 730256, University Park, Pennsylvania, USA; 5 Pennsylvania Department of Health 6616, Harrisburg, Pennsylvania, USA; UMR Processus Infectieux en Milieu Insulaire Tropical, Ste. Clotilde, France

**Keywords:** pangenome, zoonoses, One Health, humans, cattle, *Salmonella*

## Abstract

Increasingly, antimicrobial-resistant (AMR) *Salmonella* Dublin is a threat to human and animal health, therefore requiring a One Health approach to comprehensively understand pathogen evolution. Moreover, *S*. Dublin dissemination throughout the United States and the food supply chain is a concern for food safety and security. Here, we leveraged multi-agency biosurveillance data and genomic sequencing of *S*. Dublin strains to provide a robust analysis of its evolution across human, animal, and environmental reservoirs. This study advances our understanding of AMR *S*. Dublin, elucidates factors driving AMR emergence, and informs interventions to protect public health. In total, 2,150 strains collected between 2002 and 2023 throughout the United States from clinical bovine (*N* = 581), clinical human (*N* = 664), and environmental (*N* = 905) sources were identified. After uniform quality control, raw reads were assembled *de novo* followed by genome annotation and characterization of plasmids, antimicrobial resistance genes, and virulence factors. Strain relatedness was evaluated using a core genome maximum-likelihood phylogeny and pairwise core genome single-nucleotide polymorphism (SNP) differences. We identified the highest prevalence of drug-specific antimicrobial resistance genes and multidrug resistance plasmid, IncA/C2 (*P* < 0.001), in bovine clinical strains, which also had the greatest genetic diversity. Despite source-dependent differences in antimicrobial resistance gene frequency and types, 72% of *S*. Dublin strains in our study differed with at least one other strain by 20 or fewer SNPs. This high degree of genomic similarity highlights the potential for cross-transmission between humans, animals, and the environment and underscores the importance of considering strain source when assessing and monitoring antimicrobial resistance.

**IMPORTANCE:**

*Salmonella* Dublin is a zoonotic, sometimes foodborne, pathogen that causes severe illness in cattle and humans. Our study takes a One Health approach to understanding genetic differences in strains within and between different reservoirs in the United States. We identified differences in antimicrobial resistance potential and genome content between clinical bovine, clinical human, and environmental strains. Nonetheless, the U.S. population of *S*. Dublin is highly related and diverges minimally over time and geography. These findings highlight the importance of the One Health framework when combating zoonotic antimicrobial-resistant pathogens like *Salmonella* Dublin.

## INTRODUCTION

*Salmonella enterica* subsp. *enterica* serovar Dublin (*S*. Dublin) is a bovine-adapted zoonotic pathogen, capable of causing invasive disease in both cattle and human hosts. In cattle, *S*. Dublin has been reported as the most common serovar isolated from clinical case submissions and the second most common serovar from nonclinical submissions (1). While, like other nontyphoidal *Salmonella*, transmission is primarily fecal-oral, cattle-to-cattle and cattle-to-human transmission may also occur via contaminated saliva or milk (2). In cattle, *in utero* transmission has also been reported and can lead to abortion (3). Disease manifestation varies but is often most severe in calves, where infections are invasive, leading to high rates of septicemia, respiratory disease, and death. Calves that survive are more likely to become subclinical or asymptomatic carriers and intermittent shedders later in life. In adults, clinical disease may manifest as gastroenteritis and lead to milk production losses in lactating cattle. In those that progress to a subclinical carrier state, bacterial shedding can increase in response to environmental, physiological, or disease-related stressors, contributing to higher rates of *S*. Dublin septicemia in calves born to carriers (2, 4, 5). Taken together, the invasiveness of *S*. Dublin infections in calves, the subclinical carrier state in adults, and the associated animal and milk production losses attributable to either are significantly detrimental to dairy production. Additionally, while *S*. Dublin research focuses on dairy systems, *S*. Dublin is also a concern in beef production, having been recovered in post-harvest environmental sampling and from retail meats (6, 7). *S*. Dublin in the United States is particularly concerning, as the United States is the top beef-producing country globally (8) and among the top three for major dairy commodities (9–11).

As a zoonotic pathogen, *S*. Dublin is capable of infecting humans and other animals. Many *S*. Dublin outbreaks have been foodborne, traced to cattle-derived products such as raw milk, soft cheeses, and contaminated beef (12–15). However, one study reported increased *Salmonella* Dublin infection risk through contact with cattle, implicating *S*. Dublin as an occupational hazard for individuals working with cattle such as veterinarians and producers (16). When transmission does occur, *S*. Dublin infections in humans are particularly severe relative to other serovars, with a higher proportion isolated from blood specimens (17). Additionally, when evaluating *Salmonella* tied to meat commodities such as beef, *S*. Dublin had the third highest hospitalization-to-illness ratio relative to other serovars (18). Thus, *S*. Dublin is a significant concern for both cattle and human health.

As observed in strains isolated from both cattle and humans, the infection severity is compounded by trends of increasing antimicrobial resistance (AMR) and multidrug resistance (MDR, defined as resistance ≥3 antibiotic classes) (19). Specifically, resistance has been reported to common antimicrobials approved for treatment in either system, such as ceftiofur in cattle-derived strains (20, 21) and ciprofloxacin and ceftriaxone in humans (6, 22). Monitoring of antimicrobial resistance in pathogens relevant to human and animal health, like *S*. Dublin, is supported by the activities of the National Antimicrobial Resistance Monitoring System (NARMS) (23), allowing for integrated biosurveillance by different federal, state, and local entities throughout the United States. The increased accessibility and expansion of whole-genome sequence (WGS)–based surveillance and research throughout these entities have generated substantial publicly available data, which is a valuable resource that can be leveraged to investigate spatiotemporal pathogen evolution both within and between different pathogen reservoirs. Previous studies integrating WGS when examining *S*. Dublin in the United States have focused on specific reservoirs (cattle, human, and retail meat), on specific regions within the United States, on specific time periods, or some combination thereof (6, 24, 25). Other studies that take a more comprehensive approach often include other serovars, strains from other countries, or strains from non-cattle to non-human hosts (22, 26).

Given the significance of cattle production in the U.S., the impact of *Salmonella* Dublin zoonoses on cattle and human health due to combined antimicrobial resistance and extensive virulence, and the lack of a comprehensive comparative genomic study within a One Health framework, we leveraged the existing biosurveillance infrastructure in the United States to examine the differences in all publicly available whole-genome sequenced human, bovine, and environmental strains of *Salmonella* Dublin from the United States. We report high levels of genomic stability, regardless of spatiotemporal dynamics, with some source-dependent differences in *Salmonella* Dublin strains.

## MATERIALS AND METHODS

### Query, accession, and quality filtering

To maximize the number of strains included in our study, we queried the NCBI Pathogen Isolates Browser (27) for all *Salmonella* Dublin using the search terms outlined in Table S1 (date: 05/01/2024). From the exported filtered query table, the strains with empty “Host” entries were assessed with the associated strain metadata to assign host attribution. In addition to the NCBI query, *Salmonella* Dublin strains were identified with metadata obtained through collaboration with NARMS. To account for potential overlap, duplicate strains were removed based on Sequence Read Archive (SRA) (28) accession, BioSample (29), or PulseNet strain identifiers. Combined and deduplicated, the NCBI Pathogen Isolates Browser query and NARMS database sources yielded 2,248 putative *Salmonella* Dublin strains from public databases. Twenty-four additional *Salmonella* Dublin strains from an ongoing study with the North Dakota State University Veterinary Diagnostic Lab at the time of the query were also included.

Raw sequence reads were downloaded from the SRA database, during which additional previously unidentified duplicates were removed. Strains sequenced on only Illumina short-read platforms were retained. Raw read quality was assessed using seqkit (v2.11.0) (30) and FastQC/MultiQC (v0.11.0/1.14) (31, 32). For read trimming and filtering, strains were binned into four groups based on max read length (≤ 101 bp, 149–151bp, 240–251bp, and 300–301bp). Trimming was performed using Trimmomatic (v0.39) (33) with default parameters. Where relevant, additional trimming was performed to remove Illumina Nextera or TruSeq adapters.

To account for potential sequence contamination, trimmed paired reads were processed through Kraken2 v2.1.3 (34) using the standard database with three minimum hit groups. `extract_kraken_reads.py` from KrakenTools (35) was used with `--include-children` to filter all non-*Enterobacteriaceae* reads from forward and reverse paired files. For strains with fewer than 90% reads assigned to *Enterobacteriaceae* family, if another family accounted for more than 20% of reads, they were excluded from the study. Sequencing depth on the remaining trimmed, filtered reads was calculated, and strains under 30× coverage were removed from further analysis.

### Genome assembly and quality control

Paired, trimmed*, Enterobacteriaceae-*filtered reads were assembled *de novo* with Unicycler (v0.5.0) (36) in normal mode. Genome quality, completeness, and contamination were evaluated with QUAST (v5.2.0) (37) and CheckM (v1.2.2) (38) using the lineage workflow. Assembly quality was assessed based on G + C% content, N50, total length, and contig number. Based on *Salmonella* genome characteristics, G + C% content was expected to be near 52% and the total length near 4.8Mbp. N50 was required above 5 kbp, and the number of contigs was less than 300.

### *In silico* serotyping and multi-locus sequence typing

Dual *in silico* serotyping using Seqsero2 (v1.2.1) (39) and SISTR (v1.1.1) (40) was performed. The MLST for each strain was determined using mlst (v2.23.0) (41) with PubMLST (42) *Salmonella enterica* Achtman scheme (43), including seven housekeeping genes *aroC*, *dnaN*, *hemD*, *hisD*, *purE*, *sucA*, and *thrA*. Strains not concordantly serotyped as *S*. Dublin by two or more of the following methods were excluded: SeqSero2 serotype, SISTR serotype, or SISTR cgMLST serotype.

Primary metadata for all strains was standardized for collection year, collection region defined by US Department of Health and Human Services (HHS) regions, and source (clinical human, clinical bovine, or environmental). This, combined with individual accession details, serotyping, and assembly quality results for the final data set, is outlined in Table S2. All strains excluded are outlined in Table S3.

### Strain characterization and pangenome analysis

All statistical analysis and data visualization not otherwise described was performed in RStudio (v4.4.0) (44) with additional packages *tidyverse* (2.0.0) (45) and *ggpubr* (v0.6.0) (46). Unless otherwise stated, *P* < 0.05 was considered statistically significant in all analyses.

All strain genomes were profiled for virulence genes and plasmids using ABRicate (v1.0.1) (47), with the Virulence Factor (48) and PlasmidFinder (49) databases, respectively. Differences in plasmid prevalence by source were determined with a pairwise proportion test for each plasmid identified in at least five strains for all sources. *P*-values were adjusted for multiple comparisons with the Bonferroni correction.

Genome annotation was performed with Prokka (v1.14.6) (50) with `kingdom`, `genus`, and `species` flags as “Bacteria,” “Salmonella,” and “enterica,” respectively. Additional screening for antimicrobial resistance genes (ARGs), point mutations, and curated gene sets relevant to *Salmonella* was performed with AMRFinderPlus 3.12.8 using database version 2024-07-22.1 and a 90% coverage requirement. We examined the variance of drug-related AMR profiles across strain sources by performing a pairwise PERMANOVA with *pairwiseAdonis* (v0.4.1) (51) in R using distance matrices generated with *vegan* (52). *P*-values were adjusted for multiple comparisons with the Bonferroni correction. Differences in specific ARG prevalence by source were determined with a pairwise proportion test for each ARG identified in at least 5% of all strains. *P*-values were adjusted for multiple comparisons with the Bonferroni correction.

Pangenome analysis and core genome alignment were performed on annotated assemblies using Roary (v3.13.0) (53) and MAFFT (v7.525) (54) with default parameters. As in our examination of AMR profile variance across strain sources, we performed a pairwise PERMANOVA with all annotated genes, adjusting *P*-values for multiple comparisons with the Bonferroni correction.

With *pagoo* (v0.3.18) (55) in RStudio (v4.4.0) (44), genes were categorized as core, shell, or cloud genes based on their prevalence across genomes. Fraction thresholds for each were set as follows: core ≥0.95, 0.95 > shell ≥ 0.15, and cloud < 0.15 (53). To better characterize source-based differences in core, shell, and cloud genes, genes unique to each category by source were functionally annotated using the Database of Clusters of Orthologous Genes (COGs) (56).

Pangenome openness was determined using a power law function Δ*n* = κ*N*^-α^ derived from Heap’s law (*P* = kN^λ^) (57), where Δ*n* is the number of new genes, *N* is the number of new genomes, and κ and α are fitting parameters. When α > 1, the pangenome is considered closed, and if α < 1, the pangenome is considered open (58).

To examine the differences in pangenome openness by source, the methods were adapted from Cohn et al. (59). In brief, strains were subset by source for generation of gene presence-absence matrices with Roary. In R, 100 randomly generated subsets of 30 strains from each source were examined with the power law function in *pagoo*. Variance in grouped α values was evaluated using a one-way ANOVA with post-hoc Tukey’s Honest Significant Difference test to determine significance.

### Phylogenetic relationships and single-nucleotide polymorphism analysis

ModelTest-NG (v0.2.0) (60) was used to determine the optimal nucleotide substitution model for the core genome alignment generated by Roary. Tree construction was performed using IQ-TREE (61) with UFBoot (62) for bootstrap approximation using 1,000 bootstrap replicates, the `-bnni` option to account for potential branch support overestimation, and the GTR + I + G4 model (general time reversible model with invariant sites and four gamma distribution rate categories) for nucleotide substitution. The phylogeny was visualized with iTOL (Interactive Tree of Life) (63).

Single-nucleotide polymorphism (SNP) differences were examined using snp-dists (v0.8.2) (64) to generate a matrix of pairwise SNP differences between all 2,150 strains. The same strain and redundant comparisons were excluded. All downstream analysis was performed in R. Relationships were visualized using *chorddiag* (v0.1.3) (65).

## RESULTS

### Refinement and final data set

Of the initial 2,272 strain data set, 2,252 strains met sequencing platform and raw read quality thresholds. Ten additional duplicates were identified during accession, and five strains were sequenced on the PacBio platform and therefore excluded from downstream analysis. Due to either too many ambiguous bases, low average quality score, poor-quality reverse reads, or some combination thereof, five additional strains did not meet quality inclusion criteria and were excluded. Six strains were excluded due to >20% lytic Jerseyvirus phage contamination identified in Kraken2. An additional 24 strains were excluded for failing to meet sequencing depth criteria of 30× following taxonomic filtering.

**Fig 1:**
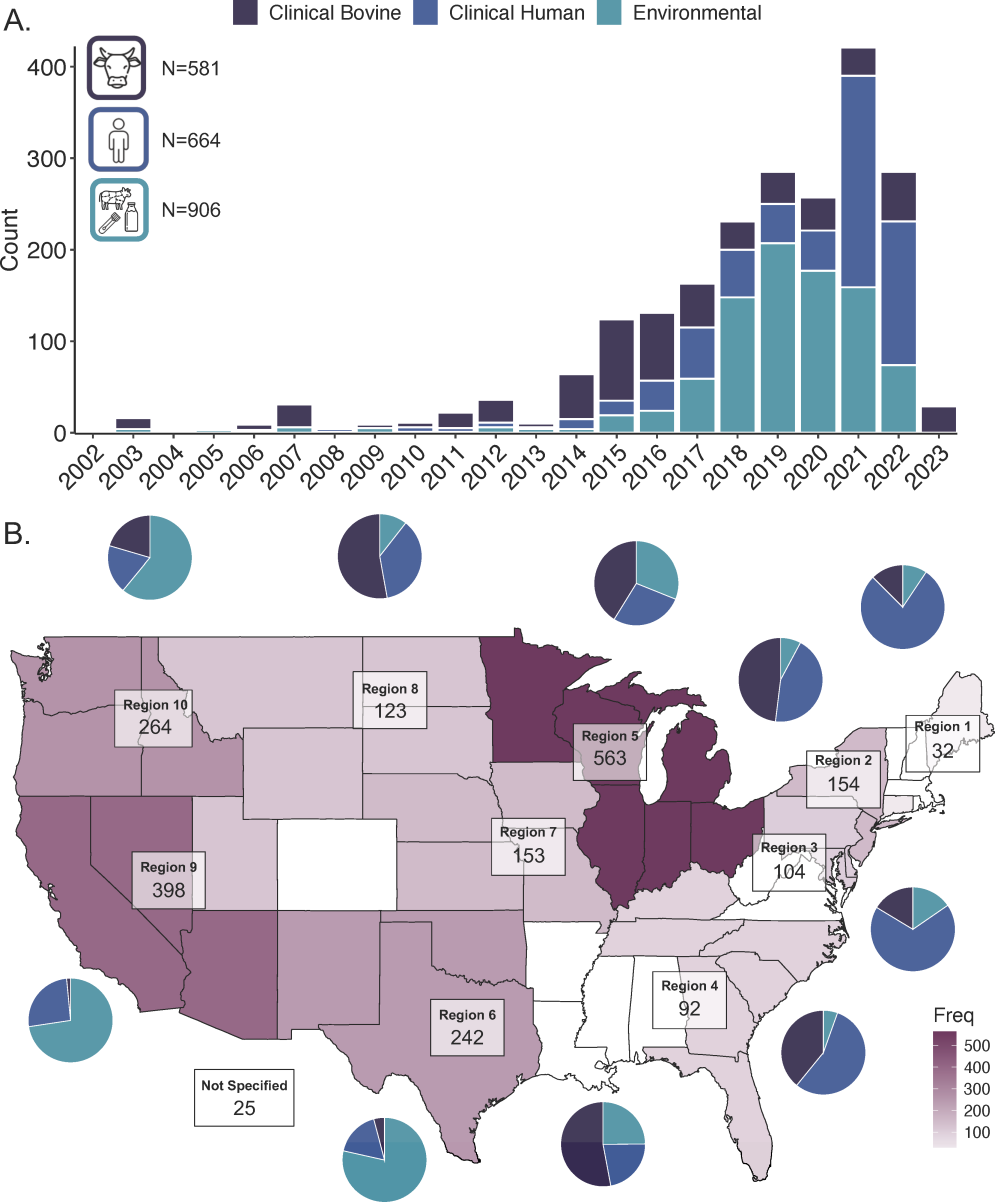
Spatiotemporal and source distribution of 2,150 *Salmonella* Dublin strains. (A) Strain source by collection year (B) strain counts and source composition by US Department of Health and Human Services (HHS) region. Corresponding regions for source composition plots are arranged clockwise: Region 10, 8, 5, 2, 1, 3, 4, 7, 6, and 9. The map was generated using R packages, maps, tidylog, tidyverse, and ggpubr. The code is available at https://github.com/sophiakenney/compare_sdublin/blob/main/R/script/plotmeta.R.

All but three genomes were successfully assembled with Unicycler, resulting in 2,219 strains that met all genome assembly quality inclusion criteria. Of these, 69 were identified as non-Dublin serotypes using SISTR and SeqSero2, with the majority (*N* = 39) typed as the Montevideo serotype. With non-Dublin strains excluded, a final set of 2,150 strains formed the basis of this analysis (Table S2 and Fig. 1). All excluded strains are outlined in Table S3.

### Final data set traits

Most strains were environmentally derived from food-associated and environmental swab samples (*N* = 906). Clinical human (*N* = 664) and clinical bovine (*N* = 581) strains were isolated from blood, urine, feces, and various tissue samples. Strains were collected from 2002 to 2023 and span all U.S. Department of Health and Human Services (HHS) regions (Fig. 1). Only twenty-five strains were uploaded to SRA with “USA” as the sole location descriptor.

All strains serotyped as *Salmonella* Dublin met genome quality inclusion criteria. Mean G + C% content, genome size, and N50 were 52.15%, 4.8 Mbp, and 295 kbp, respectively (Table S2). The mean contig number was 53. As determined by CheckM, per-sample contamination averaged 0.10%, and completeness averaged 99.64%. Most strains (2,119/2,150; 98.6%) were classified as sequence type 10 (ST10) as expected. The remainder were classified as ST2829 (*N* = 1), ST3734 (*N* = 3), ST4030 (*N* = 1), ST4619 (*N* = 1), ST4654 (*N* = 1), ST7796 (*N* = 7), and ST8054 (*N* = 1). Each non-ST10 sequence type varied by only one allele from among *hisD*, *hemD,* or *sucA*. Sixteen strains were unable to be assigned a sequence type due to a missing or untypeable allele (*N* = 10) or an approximation of the correct allele (*N* = 6) (Table S2).

### Virulence potential is conserved regardless of source

Across all strains, 116 distinct virulence genes were identified, encoding primarily functions of fimbrial adhesion, iron uptake, magnesium uptake, non-fimbrial adhesion, and effector delivery. Most genes (*N* = 99) were present in at least 99% of strains (Table S4). The least common includes *sseK2* (*N* = 1 strain), *gogB* (*N* = 3 strains), *sspH2* (*N* = 41), and *sseI/srfH* (*N* = 1709). There was no relationship between the presence of less common genes and strain source. The most common genes were related to effector delivery via type III secretion systems (Table 1). Relevant to *S*. Dublin pathogenesis and virulence, at least one of the *spv* operon genes *spvB*, *spvC*, and *spvR* was present in 1,925 (89.5%) strains (Table S4).

**TABLE 1:** Virulence gene counts in *Salmonella* Dublin by category

Category	Genes (*N*)
Fimbrial adhesion	17
Non-fimbrial adhesion	3
Macrophage-inducible gene	1
Type III secretion system	78
Intracellularly active toxin	3
Apoptosis	1
Iron uptake	9
Magnesium uptake	2
Gifsy-2 related	2

### Antimicrobial resistance varies by source

Forty-nine unique ARGs were identified across all strains via AMRFinderPlus. Within strains, ARG prevalence ranged from 1 to 10 ARGs per strain (Table S5). Most ARGs were associated with specific drug resistance (*N* = 34), followed by metal (*N* = 13) and biocide (*N* = 2) resistance (Table 2). Two efflux-associated genes, *mdsA* and *mdsB*, were present in all or nearly all strains. Drug resistance genes spanned those conferring resistance to aminoglycoside, beta-lactam, macrolide, phenicol, quinolone, quinolone/triclosan, sulfonamide, tetracycline, and trimethoprim classes. Overall prevalence of metal resistance genes to copper, gold, tellurium, or mercury varied widely (0.05%–99.95%) (Table S5).

**TABLE 2:** Antimicrobial resistance gene prevalence in *Salmonella* Dublin by type, class

Type	Class	Gene	Strains (*N*)
Drug	Aminoglycoside	*aph (6)-Id*	152
		*aph(3'')-Ib*	217
		*aph(3')-Ia*	9
		*aac (3)-IId*	1
		*aph(3''*)	1
		*aadA1*	1
		*aadA2*	2
	Beta-lactam	*blaCMY-2*	265
		*blaTEM*	35
		*blaCMY*	62
		*blaTEM-1*	10
		*blaTEM-31*	1
	Efflux	*mdsB*	2,150
		*mdsA*	2,149
	Macrolide	*acrB_R717Q*	1
	Multidrug	*ramR_T50P*	24
		*ramR_G25A*	2
		*ramR_T18P*	2
	Phenicol	*floR*	18
		*catA1*	2
	Quinolone	*gyrA_S83F*	145
		*gyrA_D87N*	334
		*gyrA_D87G*	43
		*gyrB_S464F*	12
		*gyrA_S83Y*	79
		*gyrA_D87Y*	62
		*gyrB_E466D*	15
		*qnrB19*	6
		*gyrA_A119E*	1
		*parC_S80I*	2
	Sulfonamide	*sul2*	207
		*sul1*	2
	Tetracycline	*tet(A*)	89
	Trimethoprim	*dfrA12*	1
Biocide	Quaternary ammonium	*qacE*	1
		*qacEdelta1*	3
Metal	Copper/gold	*pcoS*	1
		*golT*	2,149
		*golS*	2,149
	Mercury	*merD*	42
		*merE*	41
		*merR*	23
		*merB*	7
		*merA*	6
		*merP*	11
		*merT*	11
	Tellurium	*terD*	1
		*terZ*	1
		*terW*	1

**Fig 2:**
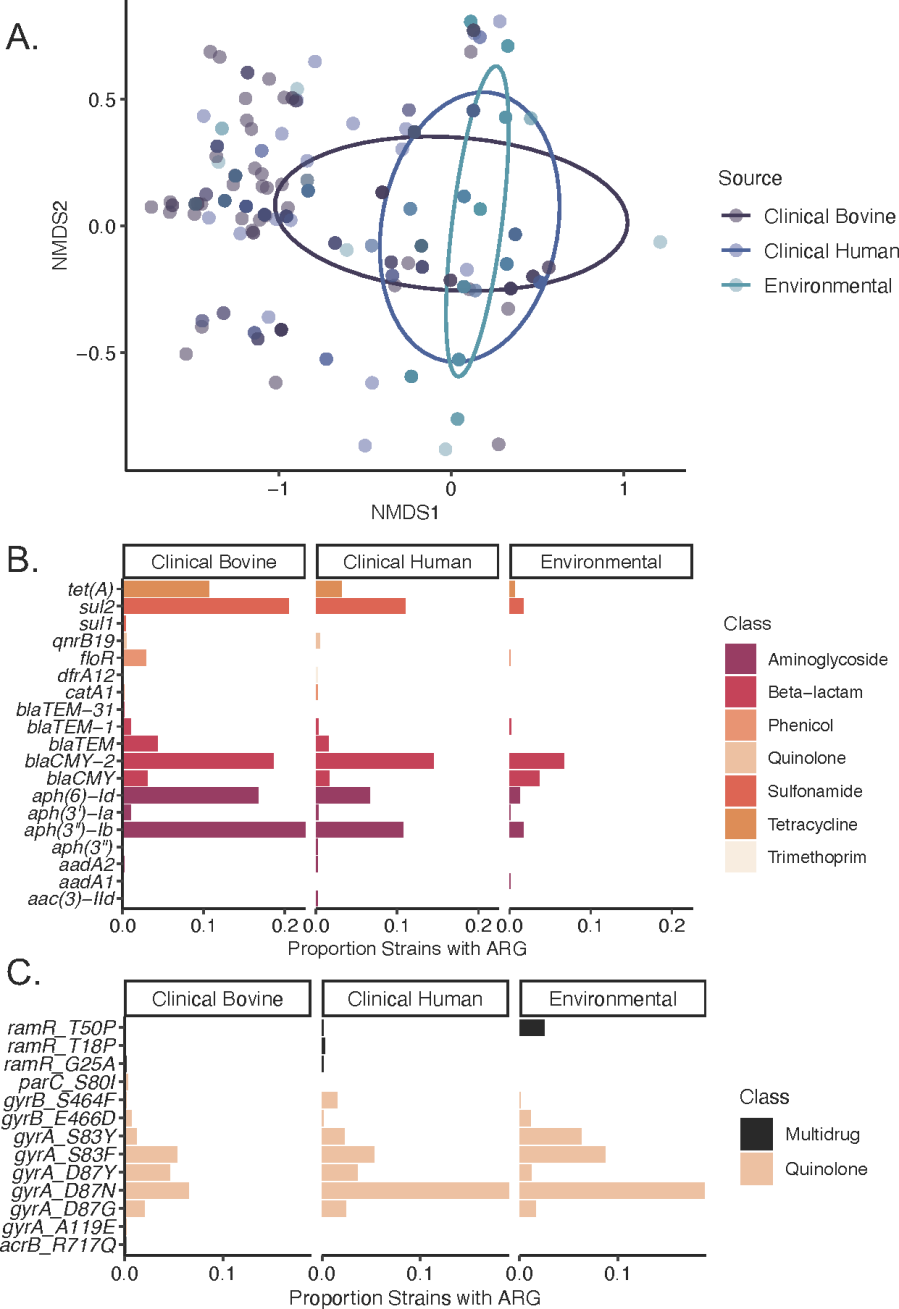
Antimicrobial resistance variance of 2,150 *Salmonella* Dublin genomes by strain source. (A) Nonmetric multidimensional scaling (NMDS) ordination of strain ARG profiles. Source indicated by color and ellipses corresponds to 95% CI, (B) drug-related ARG prevalence by source, and (C) point mutation–dependent ARG prevalence by source. Color corresponds to drug class.

When variations in ARGs between clinical human, clinical bovine, and environmental sources were examined, all pairwise comparisons were statistically significant (p-adj = 0.003) (Fig. 2A). Observed differences in specific gene prevalence (Fig. 2B) show numerically higher proportions of *tet(A)*, *sul2*, *floR*, *blaTEM*, *blaCMY-2*, *aph (6)-Id*, and *aph(3”)-Ib* in clinical strains relative to environmental strains and in clinical bovine strains relative to clinical human strains. Regarding point mutations specifically (Fig. 2C), this trend appears to reverse, with environmental strains possessing most quinolone and multidrug resistance–conferring point mutations at higher proportions relative to clinical strains. When specific gene proportions were compared, most ARGs present in at least 5% of all *S*. Dublin strains were significantly different (Table 3). For specific source comparisons, the prevalence of a few genes was not statistically significant: *blaCMY-2* (NS, bovine-human), *gyrA*[D87N] (NS, human-environmental), and *gyrA*[S83F] (NS, bovine-human).

**TABLE 3:** Antimicrobial resistance gene prevalence in *Salmonella* Dublin by source^*a*^^,^^*b*^

Gene	Clinical bovine (*N*)	Clinical human (*N*)	Environmental (*N*)	Total (*N*)	Bovine-human	Bovine- environmental	Human-environmental
*aph(3'')-Ib*	22.5% (131)	10.7% (71)	1.7% (15)	217	***	***	***
*blaCMY-2*	18.5% (108)	14.5% (96)	6.7% (61)	265	NS	***	***
*gyrA_*D87N	6.5% (38)	19.0% (126)	18.8% (170)	334	***	***	NS
*aph(6)-Id*	16.7% (97)	6.6% (44)	1.2% (11)	152	***	***	***
*gyrA_*S83F	5.3% (31)	5.3% (35)	8.7% (79)	145	NS	*	*
*sul2*	20.5% (119)	11.0% (73)	1.7% (15)	207	***	***	***

^*a*^ Bonferroni corrected *P*-values < 0.001 (***) and *P* < 0.05 (*) denote statistically significant differences in ARG prevalence between sources. NS indicates not significant.

^*b*^ Statistical significance of pairwise proportion comparisons between prevalence by sources indicated in rightmost columns. Comparisons were only performed for genes present in ≥5% of all strains.

Like increased ARG prevalence in clinical bovine strains, multidrug resistance plasmid replicon is statistically significantly (p-adjusted <0.001) more prevalent among clinical bovine strains than clinical human and environmental strains (Table 3). Prevalence of other plasmids, IncFII/S, IncX1, and ColRNAI types, also varies based on source (Table 4 and Table S6).

**TABLE 4:** Plasmid prevalence in *Salmonella* Dublin by source^*a*^^,^^*b*^

Plasmid	Clinical bovine (*N*)	Clinical human (*N*)	Environmental (*N*)	Total (*N*)	Bovine-human	Bovine-environmental	Human-environmental
IncFII(S)	91.7% (533)	87.2% (579)	84.5% (765)	87.3% (1,877)	NS	***	NS
IncX1	93.1% (541)	91.9% (610)	69% (624)	82.6% (1,775)	NS	***	***
ColRNAI	3.3% (19)	4.2% (28)	9.8% (89)	6.3% (136)		***	***
IncA/C2	14.3% (83)	4.8% (32)	2% (18)	6.2% (133)	***	***	*
Col440I	0.5% (3)	3% (20)	0.4% (4)	1.3% (27)			
IncFIA	0.7% (4)	1.2% (8)	0.8% (7)	0.9% (19)			
IncI1(alpha)	0.3% (2)	0.5% (3)	1.1% (10)	0.7% (15)			
IncFIB(pB171)	0.5% (3)	1.5% (10)	0.1% (1)	0.7% (14)			
IncFIB(AP001918)	0.5% (3)	0.2% (1)	0.6% (5)	0.4% (9)			
IncHI1A	0.9% (5)	0.3% (2)	0.1% (1)	0.4% (8)			
IncFII	0.2% (1)	0.5% (3)	0.1% (1)	0.2% (5)			
Col156	0.5% (3)	0.2% (1)	0% (0)	0.2% (4)			
ColpVC	0.3% (2)	0% (0)	0% (0)	0.1% (2)			
IncN	0.3% (2)	0% (0)	0% (0)	0.1% (2)			
IncHI1B(R27)	0.2% (1)	0.2% (1)	0% (0)	0.1% (2)			
IncFIA(HI1)	0% (0)	0.3% (2)	0% (0)	0.1% (2)			
Col(BS512)	0% (0)	0.2% (1)	0.1% (1)	0.1% (2)			
IncFII(pRSB107)	0% (0)	0% (0)	0.2% (2)	0.1% (2)			
IncHI2A	0.2% (1)	0% (0)	0% (0)	0% (1)			
IncI2(delta)	0% (0)	0.2% (1)	0% (0)	0% (1)			
pSL483	0% (0)	0.2% (1)	0% (0)	0% (1)			
Col(MG828)	0% (0)	0.2% (1)	0% (0)	0% (1)			
Col8282	0% (0)	0.2% (1)	0% (0)	0% (1)			
IncX4_2	0% (0)	0.2% (1)	0% (0)	0% (1)			

^*a*^ Bonferroni corrected *P*-values < 0.001 (***) and *P* < 0.05 (*) denote statistically significant differences in plasmid prevalence between sources. NS indicates not significant.

^*b*^ Statistical significance of pairwise proportion comparisons between prevalence by sources indicated in right most columns. Statistical comparisons were not performed for all plasmids due to small sample sizes. This is indicated by empty cells.

### Strain reservoir influences pangenome composition and openness

**Fig 3:**
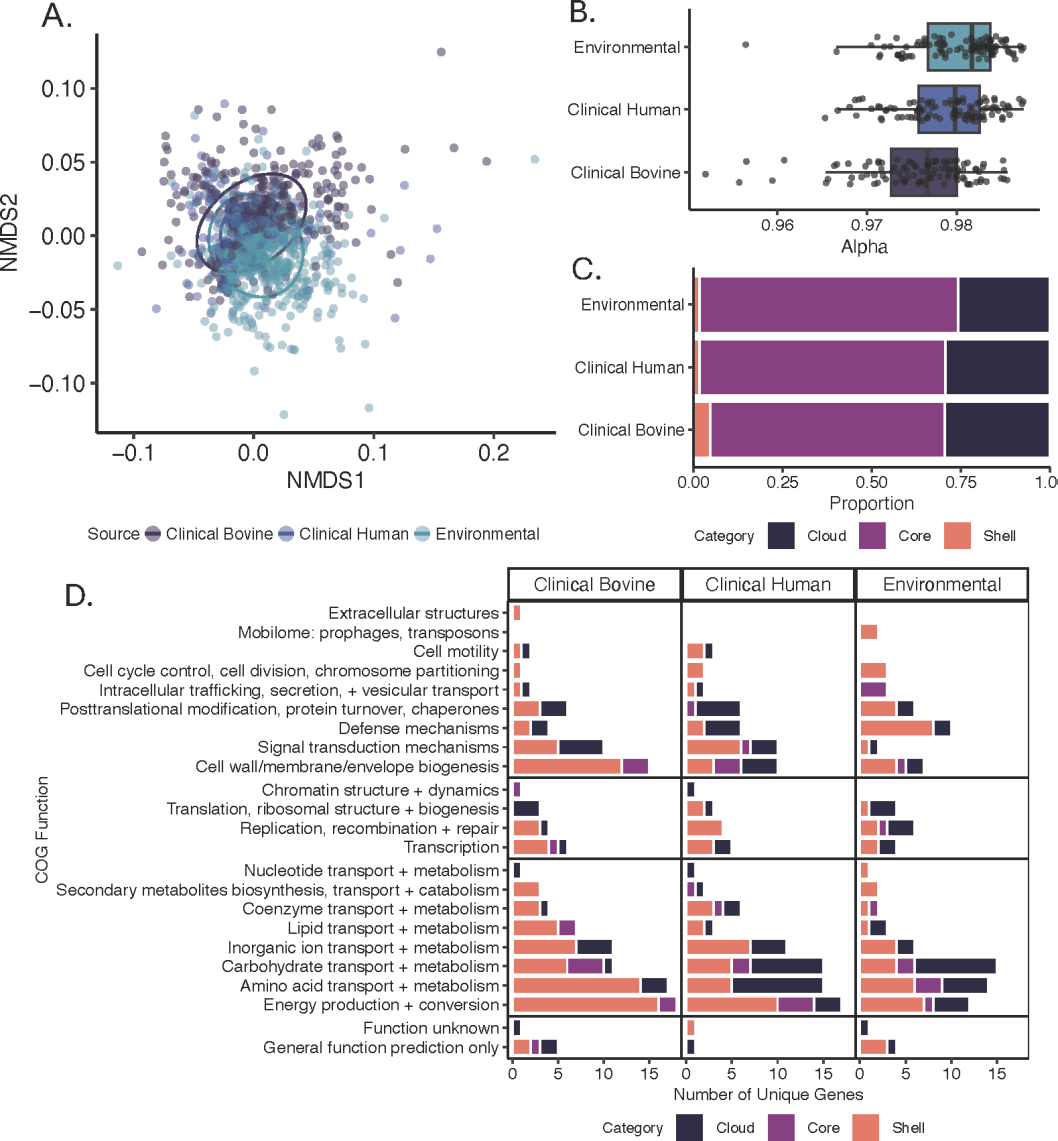
*Salmonella* Dublin pangenome varies by source. (A) Nonmetric multidimensional scaling (NMDS) ordination of strain-annotated genome profiles. Source indicated by color and ellipses corresponds to 95% CI. (B) Permutation alpha distribution by source. (C) Relative pangenome composition (core, shell, and cloud genes) by source. (D) Functional annotation of genes unique to each source and gene category. Functional category groups ordered top to bottom as follows: cellular processes and signaling, information storage and processing, metabolism, and poorly characterized. Source and/or gene category is indicated by color.

The pangenome for all strains was comprised of 8,394 total genes: 4,330 core, 2,701 shell, and 1,363 cloud. In the PERMANOVA of all annotated genes, statistically significant differences were observed between all groups (*P* = 0.003) (Fig. 3A; Table 5).

**TABLE 5:** *Salmonella* Dublin core genome summary counts by source and gene category

	Clinical bovine	Clinical human	Environmental	All strains
Core genes (*N*)	4,384	4,424	4,378	4,330
Shell genes (*N*)	1,417	1,404	1,366	2,701
Cloud genes (*N*)	868	816	597	1,363
Total genes (*N*)	6,669	6,644	6,341	8,394

When considered together, the all-strain pangenome was open (alpha = 0.8793). When strains were grouped by source (clinical bovine, clinical human, and environmental), alpha values increased towards 1 (Fig. 3B), suggesting less variability within each source. This difference in pangenome openness between sources was statistically significant (p-adj < 0.001). Notably, environmental strains exhibited the highest proportions of core genes (72.6%) and the lowest cloud and shell genes (Fig. 3C). In contrast, clinical bovine strains had the lowest proportion of core genes (65.7%) and the highest proportions of accessory (cloud and shell) genes.

When comparing differences in source-grouped pangenomes, not all genes were successfully functionally annotated, primarily because they were nonspecific hypothetical proteins or absent from the COG database. Of successfully annotated, source-specific genes (Fig. 3D), most functional categories were conserved across sources. Only a few differences in source-specific genes were present for the following categories: an extracellular structure gene in clinical bovine strains, chromatin structure and dynamics genes in clinical bovine or human strains, and mobilome genes in environmental strains. Though distribution over gene categories (core, shell, and cloud) varied, the number of source-specific genes for metabolism, information storage and processing, and cellular processes and signaling was largely consistent.

### Phylogeny and core genome single-nucleotide polymorphisms

**Fig 4:**
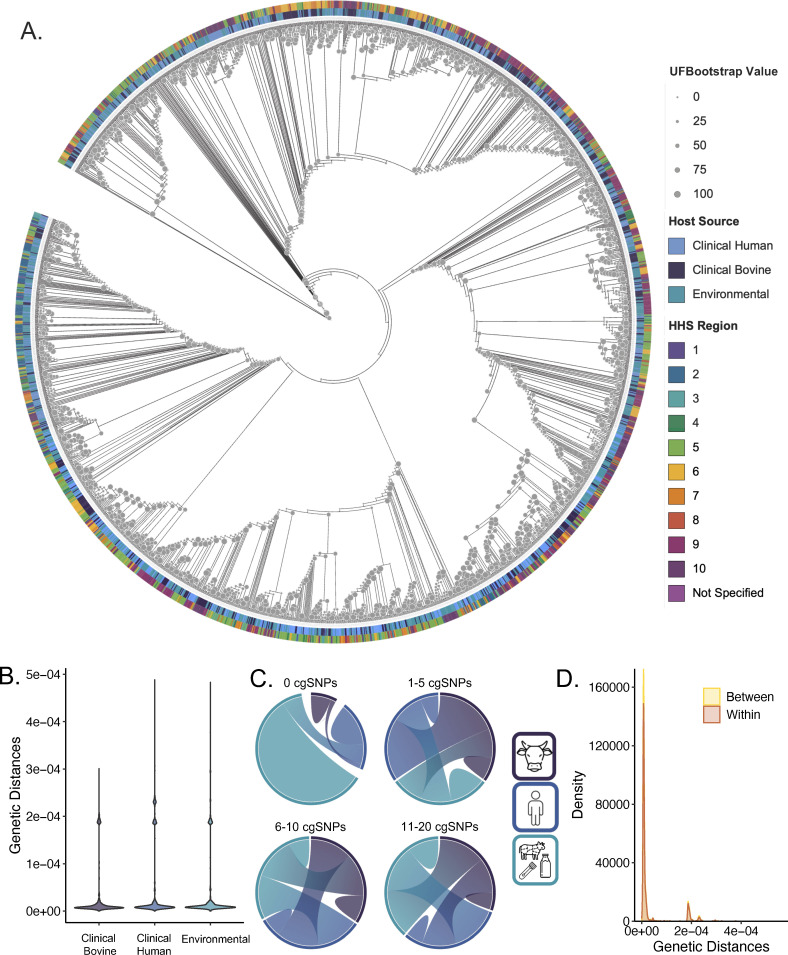
Relatedness of *Salmonella* Dublin from cattle, human, and environmental sources. (A) All strain core genome cladogram with host and HHS region denoted by outer rings and UFBootstrap value indicated by node size. (B) Distribution of genetic distances between all pairwise strain comparisons within source. (C) Chord diagram of pairwise core genome SNP (cgSNP) differences between sources (left: strains with 0, between 0 and 5, between 5 and 10, and between 10 and 20 cgSNP differences). Chord width corresponds to the number of pairs between sources where wider bands indicate more pairs. (D) Density plot of genetic distances between all pairwise strain comparisons within (orange) and between (yellow) sources.

Overall, all strains were highly related (Fig. 4B and D) with most branch lengths in the phylogenetic tree being near zero. The longest and mean branch lengths were 0.0017 and 0.0000012, respectively. Strains were distributed independent of source, collection year, and collection region throughout the tree (Fig. 4A). Hierarchical clustering revealed three primary clusters of strains, wherein strains were once more distributed independent of source, collection year, or region. While this data set was primarily comprised of MLST ST 10 strains, non-ST 10 strains were present in all but cluster 1 which only contained two strains. In cluster 2, the largest cluster, most strains were ST 10 (*N* = 1974), but non-ST 10 strains included ST 4619 (*N* = 1), ST 7769 (*N* = 7), and ST 8054 (*N* = 1). Similarly, cluster 3 was comprised primarily of ST 10 (*N* = 143) with non-ST 10 strains including ST 2829 (*N* = 1), ST 3734 (*N* = 3), ST 4030 (*N* = 1), and ST 4654 (*N* = 1) (Table S2).

Regarding pairwise comparisons, among all strains, 1,996 differed from at least one other strain by ≤20 core genome SNPs (cgSNPs). Of these, 1,660 differed from at least one other strain by ≤10 cgSNPs. Of these, 1,159 differed from at least one other strain by ≤5 cgSNPs. Finally, 397 strains differed from at least one other strain by 0 cgSNPs. Most (72%) pairwise strain comparisons revealed differences of only 20 cgSNPs or fewer. Source was a driver of relatedness at 0 cgSNP differences, with most strains having no cgSNP differences with another strain within the same reservoir. Some environmental strains did not differ from clinical human strains, while very few clinical bovine strains did not differ from environmental or clinical human strains. At higher cgSNP difference counts, however, strain relatedness was independent of source.

## DISCUSSION

*Salmonella* Dublin is an increasingly antimicrobial-resistant zoonotic pathogen, posing a significant threat to both human and animal health. Understanding its evolutionary history and trajectory requires a One Health approach that considers transmission between reservoirs, such as cattle and humans, particularly through interconnected food systems. Positive selection for virulence and antimicrobial resistance in one sector can impact others, highlighting the need for a comprehensive approach. Existing interagency biosurveillance infrastructure, specifically NARMS, provides valuable data sets that can be leveraged for comparative genomic studies to examine potential source- and spatiotemporal-dependent selection in *S*. Dublin. Our study leveraged all publicly available *Salmonella* Dublin strains collected from cattle, human, and bovine-associated environmental sources in the United States to evaluate population traits and relationships. Though we found a high level of genomic similarity across strains, independent of collection source, year, or location, we identified source-dependent differences in ARG prevalence, plasmid prevalence, and pangenome openness. Collectively, while these findings point to possible reservoir-dependent differences in selective pressure and genome stability that warrant further study, they also highlight the need for a One Health emphasis in understanding the interconnected evolution of *Salmonella* Dublin.

Elevated AMR and MDR in *S*. Dublin are generally a U.S.-specific phenomenon when compared to *S*. Dublin strains globally (26, 66). Consistent with these findings, a range of ARGs were identified across all strains, and genes conferring resistance to tetracycline, sulfonamide, and beta-lactam class drugs were more prevalent in clinical bovine strains relative to other strain sources. Among elevated beta-lactamase genes, *blaCMY* and variants were most prevalent. The presence of *blaCMY* is associated with third-generation cephalosporin resistance (67), a beta-lactam class deemed highest priority and critically important by the World Health Organization (68). Moreover, this class is used to treat invasive *Salmonella* Dublin infections severe enough to warrant antimicrobial intervention in neonatal calves as well as children (69, 70). Additionally, *floR*, associated with phenicol resistance, was most prevalent in clinical bovine strains. Though florfenicol is not effective for treating *Salmonella* in cattle, it is approved for treating calf pneumonia; thus, resistance selection may arise from florfenicol treatment in *S*. Dublin cases passing as other respiratory diseases (71).

Different chromosomal point mutations and plasmid-encoded ARGs have been previously reported in U.S. strains (24–26). In this study, we found that quinolone resistance-conferring mutation prevalence was highest in environmental strains. Though quinolones are not approved for use in cattle, ciprofloxacin is a primary treatment for invasive *Salmonella* infections in adults (70), and many of the environmental strains in this study were obtained from post-harvest food supply chain samples. Regarding plasmids, many plasmid-encoded ARGs, and thus the generally elevated AMR of U.S. strains, are linked to the circulating multidrug resistance IncA/C2 plasmid (26). We observed that the prevalence of this plasmid was highest in clinical bovine strains, followed by clinical human strains, and lowest in environmental strains, mirroring the trend observed in non-mutation ARGs. Given that (1) the fitness cost of chromosomal point mutation AMR acquisition is higher relative to that of mobile genetic elements (MGEs) like plasmids and (2) MGE acquisition is somewhat dependent on exposure to diverse microbial populations and environments harboring different MGEs, while point mutations can be accumulated regardless, the differences in overall plasmid and point mutation prevalence between clinical and environmental strains may, in part, be explained by the diversity of microbial and physical environment exposures each reservoir is subject to (72).

Consistent with the possible role of microbial and physical environment in shaping *Salmonella* Dublin genomic evolution, we found increased genome stability across environmental strains relative to clinical strains. This difference was most pronounced between clinical bovine and environmental strains, wherein clinical bovine strains had a higher proportion of accessory genome content and environmental strains had a higher proportion of core genome content. This difference in accessory genome content may in part be attributable to the lower plasmid carriage by environmental strains, wherein plasmids account partially for the increased accessory genome content of clinical bovine and clinical human strains (73). Future studies should examine the contributions of chromosomal and plasmids to overall core and accessory genome components.

As *Salmonella* Dublin can persist both in-host and farm environments, it is reasonable to suggest that strains eliciting clinical symptoms warranting diagnostic inquiry may be more adaptable, as they must survive physical environment changes (e.g., seasonal temperatures and weather conditions), host physiological changes and defenses if in carriers, and the microbial ecological dynamics within and outside of the host (74–76). Conversely, environmental strains, while having to endure similar exposures, may be less adaptable and less capable of successfully invading and causing clinical disease, thus contributing to food supply chain introduction post-harvest via asymptomatic and otherwise healthy cattle. Moreover, once in the supply chain, many environmental exposures are more controlled due to Hazard Analysis and Critical Control Point (HACCP) systems, thereby requiring less diverse adaptation (77–79).

Where clinical bovine and environmental strains diverge across these comparisons, clinical human strains consistently represent a middle ground. This may be due to exposure to clinical bovine strains on-farm or environmental foodborne strains such that the clinical human strain population represents a mix of strains. While we cannot infer transmission directionality from the data in this study, previous reports of *S*. Dublin outbreaks from food (12) or bovine contact (16) corroborate this hypothesis. Additionally, the in-between status of human strains may be tied to *Salmonella* Dublin’s need for sufficient human adaptation to cause invasive infection once transmitted. It is also possible that a level of ascertainment bias may contribute to observed differences in clinical and environmental strains (80). Strains representing clinical bovine or human sources in this data set were likely those capable of causing sufficiently symptomatic infection such that diagnosis and culture occurred. It is less likely that strains causing more mild disease, where medical treatment and culture are not pursued, are represented. Future studies examining *Salmonella* Dublin genomic evolution and adaptation should aim to not only account for different reservoirs but should also consider within-reservoir subpopulations of varying pathogenicity.

Regardless of any source-dependent differences, high genome stability overall was consistent with cgSNP differences and phylogenetic relationships. Most strains differed by fewer than 20 cgSNPs. Strains with zero cgSNP differences paired primarily within the same source. If strains differed by more than zero cgSNPs, the same consistency was not observed for source, location, or collection year. Nonetheless, all strains were highly related. This is consistent with studies conducted in other countries that, when examining native *S*. Dublin strain relatedness, revealed highly clonal populations (15, 81). As a zoonotic pathogen, the close relationships of *Salmonella* Dublin in the United States, regardless of source, underscore the need for antimicrobial stewardship in both human and animal health sectors, as positive selection for resistance in one sector has implications for resistance in the other. Additionally, this relatedness highlights the importance of food safety and biosecurity awareness, given *Salmonella* Dublin transmission routes within and between cattle and humans.

While public databases were ultimately beneficial for the scope of this study, some limitations are nonetheless inherent. Identifying potential strains meeting query inclusion criteria was dependent upon sufficient and correct metadata entry. It is possible that strains were inadvertently omitted due to having no host, source, or country listed in their metadata. Of the strains we included after our initial query, sequence quality was variable, and in some strains, we identified substantial Jerseyvirus contamination. With *in silico* serotyping, we identified numerous non-Dublin serovars that were listed as Dublin in their WGS metadata, the most common being Montevideo. For all these reasons, numerous strains from the original query were excluded, reducing the time range and final sample size. Finally, the ability to draw conclusions based on spatiotemporal data were also limited by metadata entry. For example, while some strains had month- and state-specific information, others had only year- and HHS region–specific information. Subsetting this data set by source, year, region, or some combination thereof, followed by identifying more specific collection information from WGS submitters, may be one approach for improving transmission directionality inference and allowing for meaningful epidemiological conclusions. Finally, though we filtered PacBio sequence reads from the study as they were not available for all strains, incorporating long read sequencing into pathogen biosurveillance would enhance genome resolution of complex regions and decrease overall fragmentation, thereby allowing for more comprehensive annotation and phylogenetic inference (82).

Despite the inherent limitations of publicly available sequence data, these data are an invaluable resource for elucidating pathogen evolution. Our analyses of *Salmonella* Dublin reveal a striking degree of genomic similarity among strains circulating in U.S. cattle, human, and environmental reservoirs. However, this apparent homogeneity masks differences in genomic stability and antimicrobial resistance elements, highlighting distinct evolutionary trajectories within each reservoir. These findings underscore the importance of continuous biosurveillance of *S*. Dublin across all interconnected sectors. A comprehensive One Health approach is crucial for effective *S*. Dublin management, integrating surveillance data from human, animal, and environmental sources to combat the evolving threat of this multifaceted pathogen.

## Data Availability

All relevant data and/or code are within the paper and its supplemental material or on GitHub at https://github.com/sophiakenney/compare_sdublin.

## SUPPLEMENTAL MATERIAL

Supplemental tables aem.00689-25-s0001.xlsx Tables S1 to S6.

ASM does not own the copyrights to Supplemental Material that may be linked to, or accessed through, an article. The authors have granted ASM a non-exclusive, world-wide license to publish the Supplemental Material files. Please contact the corresponding author directly for reuse.
